# PIC, a paediatric-specific intensive care database

**DOI:** 10.1038/s41597-020-0355-4

**Published:** 2020-01-13

**Authors:** Xian Zeng, Gang Yu, Yang Lu, Linhua Tan, Xiujing Wu, Shanshan Shi, Huilong Duan, Qiang Shu, Haomin Li

**Affiliations:** 1 The Children’s Hospital of Zhejiang University School of Medicine and National Clinical Research Center for Child Health, Hangzhou, China; 2 The College of Biomedical Engineering and Instrument Science, Zhejiang University, Hangzhou, China

**Keywords:** Paediatrics, Health services

## Abstract

PIC (Paediatric Intensive Care) is a large paediatric-specific, single-centre, bilingual database comprising information relating to children admitted to critical care units at a large children’s hospital in China. The database is deidentified and includes vital sign measurements, medications, laboratory measurements, fluid balance, diagnostic codes, length of hospital stays, survival data, and more. The data are publicly available after registration, which includes completion of a training course on research with human subjects and signing of a data use agreement mandating responsible handling of the data and adherence to the principle of collaborative research. Although the PIC can be considered an extension of the widely used MIMIC (Medical Information Mart for Intensive Care) database in the field of paediatric critical care, it has many unique characteristics and can support database-based academic and industrial applications such as machine learning algorithms, clinical decision support tools, quality improvement initiatives, and international data sharing.

## Abstract (summary)

**Table:** 

Measurement(s)	Demographics • Vital Signs Measurement • Medication • clinical laboratory measurement • fluid balance • length of hospital stay • survival time • microbiological information
Technology Type(s)	digital curation
Sample Characteristic - Organism	Homo sapiens
Sample Characteristic - Environment	Intensive Care Unit
Sample Characteristic - Location	China

Machine-accessible metadata file describing the reported data: 10.6084/m9.figshare.11481810

## Background & Summary

Over the past ten years, electronic health records (EHRs) have rapidly been adopted worldwide. In the US, the percentage of non-federal acute care hospitals that have adopted basic EHRs increased from 9.4% in 2008 to 83.8% in 2015^1^. In China, the healthcare system has been undergoing the New Medical Reform that has stimulated several nation-wide initiatives to encourage and assess adoption and implementation of EHRs since 2009^2^. The analysis of data contained in EHRs, which contain a large volume of structured and unstructured information regarding patient care, is a promising avenue of research for clinicians and data scientists^3–5^. However, there are several important barriers to the widespread adoption of big data in health care, including no strong incentives for its use within groups of clinicians, considerable privacy concerns, and limited interoperability^6,7^. Moreover, barriers to data sharing in healthcare have limited the usage of clinical data and largely prevented the reproducibility of published studies^8,9^.

Intensive care units (ICUs) provide care for severely ill patients who require invasive life-saving treatment. Large amounts of data are routinely collected for these critical care patients. The widely used MIMIC (Medical Information Mart for Intensive Care) critical care database has been developed for more than a decade contains comprehensive clinical data of patients admitted to the Beth Israel Deaconess Medical Centre in Boston, Massachusetts^10,11^. For many years, it has been the only freely accessible database in critical care medicine, and it supports a broad range of research areas, as evidenced by many publications^12–15^. Building upon the success of MIMIC-III, Philips Healthcare developed a multi-centre intensive care unit database called the eICU Collaborative Research Database^16^. However, all these databases have an obvious age gap in their patients. Although MIMIC-III contains data on neonates in the NICU, comprehensive paediatric patient (age from 0 to 18 years old) data are not available in it. Children are not just ‘small adults’ and often have different diseases, developmental issues, and possibly differential responses to therapies and the recovery of function when compared with adult patients in critical care^17–20^. There is vast variability in age-related differences that are all linked to the developmental stage and ability of children. Therefore, all clinical evidence should also be validated in children before being applied in paediatrics. Although there are some large-scale paediatric critical care research databases based on case registries, such as the Virtual PICU Systems (VPS) database in the US^21^ and the Paediatric Intensive Care Audit Network (PICANet) in the UK^22^, there is a lack of a freely accessible database that contains the fidelity and completeness of raw paediatric intensive care clinical data for researchers. A freely accessible paediatric-specific critical care database will stimulate more researchers to pay attention to children’s intensive care and improve the quality of critical care for children.

**Fig. 1:**
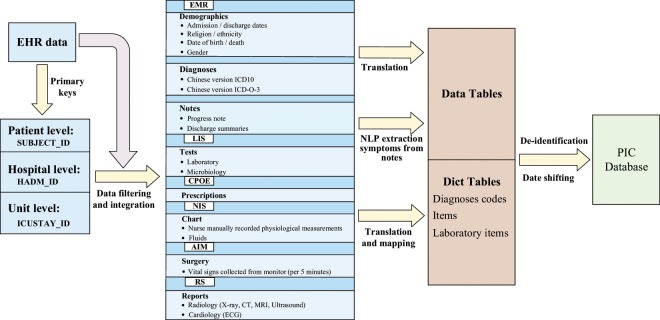
Overview of the PIC database.

Here, we report the release of the PIC (Paediatric Intensive Care) database, a freely accessible paediatric-specific critical care database (http://pic.nbscn.org). Like the widely used MIMIC database, the PIC database integrates deidentified, comprehensive clinical data of paediatric patients admitted to the Children’s Hospital of Zhejiang University School of Medicine and makes it internationally accessible to researchers under a data use agreement (Fig. 1).

The PIC database is notable for the following reasons:

- It is publicly and freely available to researchers worldwide as a paediatric critical care supplement to the MIMIC-III
- It encompasses a very large population of paediatric patients and spans almost a decade;
- It contains high temporal resolution and high-fidelity clinical data;
- It is the first freely accessible English-Chinese bilingual clinical database.

## Methods

The PIC database was populated with data that had been acquired during routine hospital care The Children’s Hospital, Zhejiang University School of Medicine. This children’s hospital, with more than 1900 beds, is the largest comprehensive paediatric medical centre in Zhejiang Province and the Chinese National Clinical Research Centre of Child Health. There are more than 3 million outpatient and emergency visits per year in this children’s only medical center. It also accepts critical paediatric patients who referred from lower level hospital across 11 cities of Zhejiang province. It has 119 critical care beds in 5 intensive care units: general ICU, paediatric ICU (PICU), surgical ICU (SICU), cardiac ICU (CICU) and neonatal ICU (NICU). The clinical data of patients admitted to any of the ICUs between 2010 and 2018 was used to construct the PIC database. This project was approved by the Institutional Review Board of the Children’s Hospital, Zhejiang University School of Medicine (Hangzhou, China). The requirement for individual patient consent was waived because the project did not impact clinical care, and all protected health information was deidentified.

## Database Development

Data were extracted and downloaded from several information systems in the hospital, including the following:

- Hospital electronic medical record system
- Laboratory information system
- Computerized physician order entry system
- Nursing information system
- Anaesthesia information management system
- Reporting system of different examination departments (radiology, ultrasound, ECG, pathology, *et al*.).

The core database schema followed the classic MIMIC-III database with some adjustments to adapt it for local Chinese data. This will help researchers who have experience with MIMIC to easily understand and utilize the PIC and to compare it with the MIMIC database.

All patients who transferred to any of the ICUs and had records in the hospital medical record system between 2010 and 2018 were included in this project. Based on these data, three core tables were created to describe the patients (PATIENTS), admissions (ADMISSIONS) and ICU stays (ICUSTAYS). The primary key of the three core tables was used to index all other clinical data. SUBJECT_ID in the PATIENTS table is a unique identifier that specifies an individual patient, HADM_ID in the ADMISSIONS table is the encounter number that uniquely identifies a particular hospitalization for patients who might have been admitted multiple times, and ICUSTAY_ID in the ICUSTAYS table refers to a unique admission to an intensive care unit. Each SUBJECT_ID has one or more related HADM_IDs, and each HADM_ID can have one or more related ICUSTAY_ID.

After collecting the original data, we performed post-processing and integrated medical records to ensure that each patient had relatively complete data. Since the time spans of the data in each table from the different systems are inconsistent, we used SUBJECT_ID and HADM_ID, which appear in the ADMISSIONS, PATIENTS, and ICUSTAYS table for data integration and filtering. In parallel, we carefully checked for impossible data entries (for example, the year of the visit date was 1900), erroneous characters (for example, Chinese characters appearing as numbers), and extreme outliers in the collected data and removed or updated these data.

Following the MIMIC-III database schema, structured clinical data, which included patient demographics, medications, fluid balances, comprehensive laboratory results, and microbiological information (tests performed and sensitivities) from the patients’ entire hospital stay, not only periods in the ICU, were collected from different systems. Another specially structured type of data is time-series data, such as vital signs directly collected from monitoring devices, as there are no critical care information systems to store the high-frequency automatically collected vital signs. The PIC only used the physiological measurements manually recorded by nurses. However, the PIC integrated the vital signs collected during surgery every 5 minutes from the anaesthesia information management system. To help research to distinguish these vital data from MIMIC, we created a new table named SURGERY_VITAL_SIGNS.

To make this database widely used worldwide, the largest challenge is the language barrier. All the lab test items, medication names, examinations and diagnoses were recorded using Chinese in the original information systems. To address this problem, the PIC provided English and Chinese bilingual dictionary tables to make the data can be interpretable by most researchers. For terms with widely used standard codes, such as the ICD-10 codes for diagnostics, the English terms were based on the corresponding English version of the standard codes. However, the local ICD-10 version is an extended version with 7 characters compared to the standard 5-character ICD-10 code, and therefore, the Chinese diagnosis is more specific than the English diagnosis in the PIC. Except some of lab test items with LOINC code, other clinical terms without a standard code were translated to English and reviewed manually by authors (X.Z., Y.L. and H.L.). We keep the original Chinese terms in all the tables with corresponding English terms and will help researchers identify language issues if there are any. Such a bilingual Chinese-English clinical dictionary table also creates a data environment that can serve other potential international data sharing projects.

The free-text clinical documents and reports were also recorded in Chinese. Translating a large volume of narrative clinical documents and reports is not feasible, and automatically de-identification tools and algorithms for Chinese clinical documents is also not available. We could not release the original clinical documents at this stage. However, much important clinical information is embedded in the narrative of clinical documents, so common symptoms were extracted from clinical progress notes and discharge summaries using Natural Language Processing (NLP) technology^23^. These symptom terms and their negation status (present symptom with “+” and absence symptom with “−”) data are combined with the document recording time and published as the EMR_SYMPTOMS table in the first release of the PIC. To initially evaluate the accuracy of the NLP extraction. We randomly selected 100 notes including admission note, discharge note, progress note, transferred note, and so on from the original notes, extracted symptoms from them using NLP technology, and finally resulted in 3410 symptoms. Two authors independently checked the extracted results. The average accuracy is 91.9% (91.3%, 92.4%). Several NLP typical errors should be mentioned. First, wrong segmentation of long Chinese phrases which do not have natural word space such as English. Second, falsely negative detection both in positive and negative. Third, the complicated sentence that talk about the family symptoms or the risk of some condition were also extracted as symptoms of patient. Taking into account the accuracy of NLP, we remind researchers to use these data with caution.

For many examinations and their reports, the PIC only records an examination event without the content of the reports in this release. We will try to standardize most examination results in the future and publish it in the release of the next iteration of the database.

## Deidentification

Before data were integrated into the PIC database, it was fully anonymized by removing all HIPAA (Health Insurance Portability and Accountability Act) protected health information identifiers such as patient name, address, telephone numbers, and any other characteristic that could uniquely identify the individual in structured data sources. For free-text fields, protected health information was not extracted in accordance with HIPAA standards. When creating the dataset, patient IDs including SUBJECT_ID, HADM_ID and ICUSTAY_ID were randomly assigned a unique number, and the original identifier was not retained. As a result, the identifiers in the PIC cannot be linked back to the original, identifiable data. In addition, dates such as birth date and death date were shifted to the future in a consistent manner by the random offset (50–100 years) of each patient to maintain the interval, resulting in stays that occur sometimes between 2060 and 2120. Time of day, day of the week, and approximate seasonality (we divided 12 months into four seasons: spring: March, April, May; summer: June, July, August; autumn: September, October, November; winter: December, January, February) were conserved during the date transformation. Shift the time in such a wide range may limit the utilization of PIC in many time-sensitive studies. At the same time there are also algorithm to obscures date information to any desired granularity^24^. While, the proposed approach definitely can reduce the risk of re-identification of death children. The ethnicity of patients from ethnic minorities with a small population was not specified in the PIC. As all patients allow to be accepted by this Children’s Hospital must under the age of 18, we do not need to handle the birthday of patients who were older than 90 years old to hide their real age as MIMIC.

## Data Records

Data available in the PIC database includes laboratory measurements, charted observations during a patient’s stay, structured symptoms extracted from notes and vital signs recorded while a patient was present in the operating room. The PIC consists of 16 tables that are linked by unique identifiers^25^. Tables with a ‘D’ prefix are dictionary tables and provide a definition of the identifiers. For example, each row of LABEVENTS is associated with an ITEMID that represents the concept of the measurement, but it does not contain the actual name of the measurement. By joining LABEVENTS and D_LABITEMS on the ITEMID, the concept can be identified represented by a given ITEMID.

Table 1 provides summary descriptions of the data tables, and more detail data schema and technique document about the data tables is available on the PIC website^26^. Briefly, there are three tables used to define and track patient hospitalization: ADMISSIONS; PATIENTS; and ICUSTAYS. The three identifiers described earlier (SUBJECT_ID, HADM_ID, ICUSTAY_ID) are present in all three of the above tables. The other three tables are dictionaries for defining disease codes and items appearing in the PIC database. The remaining tables contain data related to patient care during the hospital stay, such as demographics, laboratory test results, medications, symptoms, vital sign measurements, and mortality. All the tables are distributed as a collection of comma separated value (CSV) files that can be loaded into many relational database systems.

**Table 1:** An overview of the data tables in the PIC database.

Table name	Description	Rows
ADMISSIONS	Every unique hospitalization for each patient in the database (defines HADM_ID)	13,449
CHARTEVENTS	All charted observations for the patients from the hospital database, not including bedside monitor trends and lab events.	2,278,978
D_ICD_DIAGNOSES	Dictionary of International Statistical Classification of Diseases and Related Health Problems (ICD-10) codes and International Classification of Diseases for Oncology (ICD-O-3) relating to diagnoses.	25,378
D_ITEMS	Dictionary of local codes (’ITEMIDs’) appearing in the PIC database, except those that relate to laboratory tests.	466
D_LABITEMS	Dictionary of local codes (’ITEMIDs’) appearing in the PIC database that relate to laboratory tests.	832
DIAGNOSES_ICD	Hospital assigned diagnoses, coded using the ICD-10 Chinese code and the ICD-O-3 Chinese code.	13,365
EMR_SYMPTOMS	Structured symptoms extracted from notes, including nursing and physician notes, discharge summaries and so on.	402,142
ICUSTAYS	Every unique ICU stay in the database (defines ICUSTAY_ID).	13,941
INPUTEVENTS	Calculated fluid input data every morning for each patient in ICU.	26,884
LABEVENTS	Laboratory measurements for patients from the hospital database.	10,094,117
MICROBIOLOGYEVENTS	Microbiology culture results and antibiotic sensitivities from the hospital database.	183,869
OR_EXAM_REPORTS	Contains all exams performed during the patient’s stay.	183,809
OUTPUTEVENTS	Output information for patients from the hospital database.	39,891
PATIENTS	Every unique patient in the database (defines SUBJECT_ID).	12,881
PRESCRIPTIONS	Medications ordered for a given patient	1,256,591
SURGERY_VITAL_SIGNS	Vital signs recorded every 5 minutes during the surgery	1,216,011

## Technical Validation

### Paediatric-specific data features

The PIC encompasses 13,499 distinct hospital admissions from 12,881 distinct paediatric patients (aged 0–18 years) admitted to critical care unit between 2010 and 2018. Of those patients, 468 (3.6%) had multiple hospital admissions. The mean age of the patients was 2.5 years (Q1–Q3: 0.1–3.3), 57.5% of the patients were male, and the in-hospital mortality was 7.1%. The mean hospital stay was 17.6 days (Q1–Q3: 7.0–21.0), and the mean ICU stay was 9.3 days (Q1–Q3: 0.9–9.2). The mean ICU stay was longest in the neonatal ICU, 21.6 days (Q1–Q3: 2.5–32.8), and the shortest stay was in the surgical ICU, 2.3 days (Q1–Q3: 0.8–1.6). The summary statistics and detailed patient demographics of the paediatric population in the PIC stratified by care unit are listed in Table 2.

**Table 2:** Details of the PIC patient population by the first critical care unit on hospital admission.

Critical care unit	CICU	GICU	NICU	PICU	SICU	Total
Patients, no. (%)	2583 (20.1%)	2642 (20.5%)	3137 (24.4%)	1953 (15.2%)	2566 (19.9%)	12881 (100%)
Admissions, no. (%)	2638 (19.6%)	2725 (20.3%)	3205 (23.8%)	2084 (15.5%)	2797 (20.8%)	13449 (100%)
ICU stay, no. (%)	2803 (20.1%)	2788 (20.0%)	3282 (23.5%)	2166 (15.5%)	2902 (20.8%)	13941 (100%)
Age, years, mean (Q1–Q3)	2.5 (0.5–3.1)	3.8 (0.4–6.3)	14.5 days (0.0–18.0)	3.6 (0.5–5.7)	3.2 (0.4–4.9)	2.5 (0.1–3.3)
Gender, male, % of unit stays	1391 (49.6%)	1712 (61.4%)	1984 (60.5%)	1250 (57.7%)	1680 (57.9%)	8017 (57.5%)
ICU LOS, mean days (Q1–Q3)	3.9 (0.9–4.0)	7.3 (0.9–8.9)	21.6 (2.5–32.8)	9.7 (2.0–11.1)	2.3 (0.8–1.6)	9.3 (0.9–9.2)
HLOS, mean days (Q1–Q3)	16.6 (9.0–19.2)	12.8 (3.8–16.2)	27.1 (9.6–37.8)	14.5 (4.6–16.7)	14.7 (7.0–18.7)	17.6 (7.0–21.0)
ICU mortality, %	48 (1.7%)	414 (14.8%)	236 (7.2%)	200 (9.2%)	57 (2.0%)	955 (6.9%)
Hospital mortality, %	53 (2.0%)	417 (15.3%)	239 (7.5%)	205 (9.8%)	57 (2.0%)	971 (7.2%)

CICU: cardiac intensive care unit; GICU: general intensive care unit; NICU: neonatal intensive care unit; PICU: paediatric intensive care unit; SICU: surgical intensive care unit; LOS: length of stay.

The primary diagnosis of patients in the PIC shows many unique features of paediatric populations, as shown in Online-only Table 1. The top three disease categories of the discharge codes were Q00–Q99, congenital malformations, deformations and chromosomal abnormalities (25.4%); P00–P96, certain conditions originating in the perinatal period (14.1%); and J00–J99, diseases of the respiratory system (10.3%). This is completely different from what is found in adult critical care patients and also confirms the uniqueness and necessity of the PIC.

### Comparing the PIC with the MIMIC-III

**Fig. 2:**
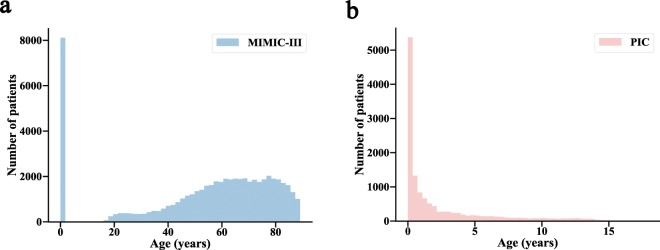
Age distributions of the MIMIC-III and the PIC databases. (**a**) MIMIC-III database. (**b**) PIC database.

Table 3 provides a comparison of the PIC with the MIMIC-III database, and Fig. 2 shows the difference in age distributions between patients in the PIC and MIMIC-III. The salient database features that are compared in Table 3 include the data source, number of records and record completeness (availability of different clinical data, physiological waveforms and outcomes). Although there are many limitations in the current release of the PIC, such as that the PIC is smaller than the MIMIC-III based on the number of ICU patients, there are no high-frequency bedside monitor data, and clinical notes are described in Chinese and are not suitable for direct release. The PIC is still notable as a supplement to the paediatric intensive care unit data lacking in the MIMIC-III and will definitely encourage more researchers to focus on paediatric critical care. Furthermore, the PIC database is under continuous development with a better critical care environment, and we hope to supplement the PIC with more and more additional data in the future, including vital signs and physiology waveforms collected at bedsides, as much structured information extracted from the clinical notes as possible, and images corresponding to any medical imaging performed during the patient’s stay.

**Table 3:** Differences of the PIC and the MIMIC-III.

	MIMIC-III	PIC
Language	English	English-Chinese bilingual
Data source	673-bed general hospital with OB/GYN	1900-bed children’s hospital
Category of critical care	CCU, CSRU, MICU, SICU, TSICU, NICU Total 77 critical care beds	CICU, SICU, PICU, NICU, GICU Total 119 paediatric critical care beds
Number of records	50,000+	10,000+
Patient age, median (Q1–Q3)	60.6 years (42.1–74.1)	0.8 years (0.1–3.5)
Vital signs	Bedside monitor generated (1 hour)	Daily nurse recorded & surgery monitor generated (5 minute)
Laboratory/clinical data	Yes	Yes
Clinician notes	Yes	Extracted symptoms from notes
Diagnoses codes	ICD-9 Codes	ICD-10 Codes and ICD-O-3 Codes
Mortality	Death recorded in the hospital and social security database	Death recorded in the hospital

### Data quality and completeness

To maintain data fidelity, the original Chinese data were kept, and most of the translated English terms were manually reviewed except the ICD-10 English terms that were considered high-quality. All data except the symptoms that were extracted from narrative documents by NLP technology had very little post-processing performed.

As different clinical information systems were implemented at different times which means the data from some information systems was not available before it implemented, the completeness of the different data tables is different. Furthermore, the vendors of nursing systems and EMR systems have changed during the time of data collected which make the data more complicated to integrated. Table 4 shows the completion of the different tables in the PIC. As a long-term development project, we are improving and upgrading various clinical information systems so that the PIC will have progressively better data quality and completeness in future versions. The source code in python to calculate the completion of different tables was released on the Github.

**Table 4:** Amount of complete data grouped by table.

Table Name	Coverage Rate	Coverage Group
CHARTEVENTS	79.7%	high
DIAGNOSES_ICD	99.4%	excellent
EMR_SYMPTOMS	6.7%	low
INPUTEVENTS	41.1%	medium
LABEVENTS	93.9%	excellent
MICROBIOLOGYEVENTS	89.7%	excellent
OR_EXAM_REPORTS	91.9%	excellent
OUTPUTEVENTS	11.7%	low
PRESCRIPTIONS	51.5%	medium
SURGERY_VITAL_SIGNS	47.7%	medium

Coverage rate was calculated at the admission level. Low (0–20%), medium (20–60%), high (60–80%), and excellent (80–100%).

### Best practice for scientific computing

Based on best practice for scientific computing^27^, the code used to build PIC was version controlled and developed collaboratively within the laboratory. The code for community to utilize and visualize the PIC dataset was hosted on GitHub. Issue tracking is used to ensure that any issues about the code and dataset can be managed and maintained. This approach encouraged and facilitated sharing of readable code and document, as well as feedback from community.

## Usage Notes

### Data access

The PIC is provided as a collection of comma separated value (CSV) files, along with example scripts to help with importing the data into the database system MySQL and PostgreSQL. As the database contains detailed information regarding the clinical care of patients, it must be treated with appropriate care and respect. Following the MIMIC protocol, researchers are required to formally request access via a process documented on the PIC website^26^ and the PhysioNet^25^. There are two key steps that must be completed before access is granted:

- the researcher must complete a recognized course in protecting human research participants that includes HIPAA requirements or obtain a GCP certification from a local institute in China.
- the researcher must sign a data use agreement, which outlines appropriate data usage and security standards, and forbids efforts to identify individual patients.

Any researchers that have been approved to access the MIMIC-III will be familiar with this process and can easily get started with the PIC. Approval requires approximately three working days. Once an application has been approved, the researcher will receive emails containing instructions for downloading the database.

### Collaborative documentation and visualization

**Fig. 3:**
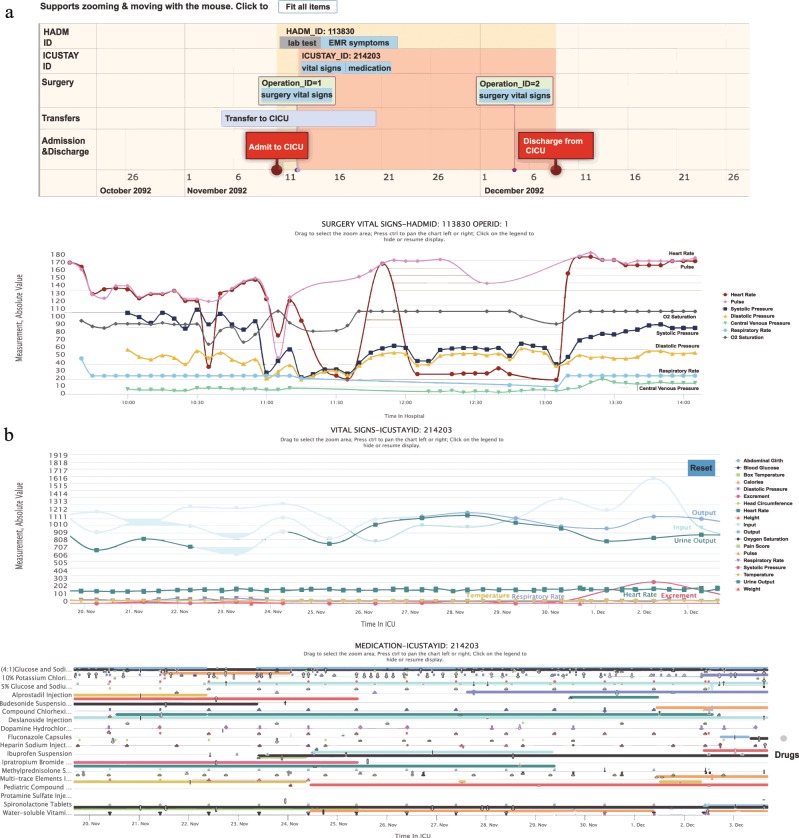
Visualization of the PIC data. (**a**) The timeline of a patient during the hospital stay. (**b**) Synchronized timelines of different data types.

The core aims for publicly developing the PIC database are to allow as much free access as possible to interested researchers and to promote secondary analysis of electronic health records. Detailed documentation is available on the PIC website and includes information regarding data access, table contents, and a schematic of the relationships between the tables in the data. The PIC is a large relational database with many different data types. This method of data storage does not visually indicate the patient’s hospitalization, nor does it quickly and easily retrieve information specific to an individual patient. It is a time-consuming process for new researchers to perform joint multiple table queries to fully understand which data are useful and available. For researchers who prefer to explore the data, we provide a website that can easily be used to explore the PIC and visualize clinical data at the patient level. As shown in Fig. 3a, admission to the hospital, surgery, transfer to the ICU and discharge from the ICU and hospital is clearly shown on a timeline. There are several data buttons for users to choose clinical data of interest. Clicking on the button will show corresponding data on a timeline. It is possible to zoom in and out on the timeline to help users better explore the details and overview of the data. We also developed a mechanism to synchronize different timelines on the webpage to help users identify relationships among different data. For example, the medicine used at a time point may explain the change in vital signs at the same time as shown in Fig. 3b. The source code for this visualization is also openly available.

To help user utilize the PIC dataset, a Jupyter Notebook was released to demonstrate how to use PIC to create a machine learning project.

## Data Availability

The code that was used to create the PIC database, calculate statistics of this paper, demonstrate a machine learning task and source code which underpins the PIC website and documentation is openly available, and contributions from the research community are encouraged: https://github.com/Healthink/PIC.

## Online-only Table

**Online-only Table 1:** Distribution of primary diagnosis by care unit for patients in the PIC database.

Critical care unit	CICU	GICU	NICU	PICU	SICU	Total
Certain infectious and parasitic diseases (A00-B99)	4 (0.2%)	117 (4.4%)	44 (1.4%)	91 (4.4%)	12 (0.4%)	268 (2.0%)
Neoplasms (C00–D48)	26 (1.0%)	198 (7.8%)	3 (0.1%)	49 (2.4%)	230 (8.1%)	506 (3.8%)
Diseases of the blood and blood-forming organs and certain disorders involving the immune mechanism (D50-D89)	0 (0.0%)	167 (6.3%)	11 (0.3%)	95 (4.6%)	10 (0.4%)	283 (2.1%)
Endocrine, nutritional and metabolic diseases (E00-E90)	4 (0.2%)	64 (2.4%)	37 (1.2%)	57 (2.7%)	22 (0.8%)	184 (1.4%)
Mental and behavioural disorders (F00-F90)	0 (0.0%)	58 (2.2%)	1 (0.0%)	2 (0.1%)	2 (0.1%)	63 (0.5%)
Diseases of the nervous system (G00-G99)	0 (0.0%)	201 (7.6%)	38 (1.2%)	274 (13.2%)	246 (8.8%)	759 (5.7%)
Diseases of the eye and adnexa (H00–H59)	0 (0.0%)	0 (0.0%)	9 (0.3%)	2 (0.1%)	8 (0.3%)	19 (0.1%)
Diseases of the ear and mastoid process (H60–H95)	0 (0.0%)	0 (0.0%)	0 (0.0%)	1 (0.0%)	3 (0.1%)	4 (0.0%)
Diseases of the circulatory system (I00–I99)	314 (11.9%)	110 (4.2%)	78 (2.4%)	153 (7.3%)	110 (3.9%)	765 (5.7%)
Diseases of the respiratory system (J00–J99)	189 (7.2%)	430 (16.3%)	173 (5.4%)	518 (24.9%)	65 (2.3%)	1375 (10.3%)
Diseases of the digestive system (K00–K93)	56 (2.1%)	184 (7.0%)	184 (5.7%)	102 (4.9%)	483 (17.3%)	1009 (7.5%)
Diseases of the skin and subcutaneous tissue (L00–L99)	0 (0.0%)	7 (0.3%)	4 (0.1%)	7 (0.3%)	19 (0.7%)	37 (0.3%)
Diseases of the musculoskeletal system and connective tissue (M00–M99)	0 (0.0%)	43 (1.6%)	14 (0.4%)	11 (0.5%)	7 (0.3%)	75 (0.6%)
Diseases of the genitourinary system (N00–N99)	0 (0.0%)	388 (14.7%)	8 (0.2%)	13 (0.6%)	12 (0.4%)	421 (3.2%)
Pregnancy, childbirth and the puerperium (O00–O99)	0 (0.0%)	0 (0.0%)	4 (0.1%)	0 (0.0%)	0 (0.0%)	4 (0.0%)
Certain conditions originating in the perinatal period (P00–P96)	34 (1.3%)	12 (0.5%)	1801 (56.2%)	11 (0.5%)	27 (1.0%)	1885 (14.1%)
Congenital malformations, deformations and chromosomal abnormalities (Q00–Q99)	1962 (74.4%)	199 (7.5%)	463 (14.4%)	74 (3.6%)	699 (25.0%)	3397 (25.4%)
Symptoms, signs and abnormal clinical and laboratory findings, not elsewhere classified (R00–R99)	30 (1.1%)	256 (9.7%)	307 (9.6%)	327 (15.7%)	352 (12.6%)	1272 (9.5%)
Injury, poisoning and certain other consequences of external causes (S00–T98)	1 (0.0%)	159 (6.0%)	16 (0.5%)	220 (10.6%)	272 (9.7%)	668 (5.0%)
External causes of morbidity and mortality (V01–Y98)	1 (0.0%)	17 (0.6%)	1 (0.0%)	39 (1.9%)	32 (1.1%)	90 (0.7%)
Factors influencing health status and contact with health services (Z00–Z99)	11 (0.4%)	4 (0.2%)	2 (0.1%)	10 (0.5%)	34 (1.2%)	61 (0.5%)
International Classification of Diseases for Oncology (M)	5 (0.2%)	31 (1.2%)	7 (0.2%)	27 (1.3%)	150 (5.4%)	220 (1.6%)
